# Climate warming and bumble bee declines: the need to consider sub-lethal heat, carry-over effects, and colony compensation

**DOI:** 10.3389/fphys.2023.1251235

**Published:** 2023-10-31

**Authors:** Sabrina A. White, Michael E. Dillon

**Affiliations:** Department of Zoology and Physiology and Program in Ecology, University of Wyoming, Laramie, WY, United States

**Keywords:** eusocial insects, temperature extremes, heat waves, thermal physiology, colony provisioning, developmental temperatures, foraging dynamics, learning

## Abstract

Global declines in abundance and diversity of insects are now well-documented and increasingly concerning given the critical and diverse roles insects play in all ecosystems. Habitat loss, invasive species, and anthropogenic chemicals are all clearly detrimental to insect populations, but mounting evidence implicates climate change as a key driver of insect declines globally. Warming temperatures combined with increased variability may expose organisms to extreme heat that exceeds tolerance, potentially driving local extirpations. In this context, heat tolerance limits (e.g., critical thermal maximum, CT_max_) have been measured for many invertebrates and are often closely linked to climate regions where animals are found. However, temperatures well below CT_max_ may also have pronounced effects on insects, but have been relatively less studied. Additionally, many insects with out-sized ecological and economic footprints are colonial (e.g., ants, social bees, termites) such that effects of heat on individuals may propagate through or be compensated by the colony. For colonial organisms, measuring direct effects on individuals may therefore reveal little about population-level impacts of changing climates. Here, we use bumble bees (genus *Bombus*) as a case study to highlight how a limited understanding of heat effects below CT_max_ and of colonial impacts and responses both likely hinder our ability to explain past and predict future climate change impacts. Insights from bumble bees suggest that, for diverse invertebrates, predicting climate change impacts will require a more nuanced understanding of the effects of heat exposure and additional studies of carry-over effects and compensatory responses by colonies.

## Introduction

Alarming global insect declines (Wagner, 2020) likely have diverse causes (Raven and Wagner, 2021), with climate warming increasingly acknowledged as a key driver (Halsch et al., 2021; Harvey et al., 2023). Strong correlations between changing climates and absence of focal species in space and time can provide compelling evidence of past climate change impacts (Parmesan and Yohe, 2003). But it has become increasingly apparent that predicting organismal responses to novel climate conditions will require a mechanistic understanding of the temperature dependence of physiological traits (Dillon et al., 2010; Somero, 2010; Huey et al., 2012). Critical thermal limits are perhaps the most common traits measured in this context; they provide an estimate of the coldest (CT_min_) and hottest (CT_max_) temperatures an organism can withstand before being incapacitated, potentially indicating permissive and inhospitable habitats (Sunday et al., 2012). It is tempting to then use CT_max_ to predict which areas will be too hot in the future given both overall climate warming and the increasing frequency of extreme heat (Perkins-Kirkpatrick and Lewis, 2020; IPCC, 2023). However, CT_max_ is remarkably invariant geographically, suggesting a limited link to existing (and future) distributions (Jørgensen et al., 2021). Further, a growing body of evidence suggests that exposure to heat well below CT_max_ can have pronounced effects (reviewed by Harvey et al., 2023; Johnson et al., 2023). And, for the diverse, abundant, and ecologically important insects that live in colonies (Hölldobler and Wilson, 1990; Hung et al., 2018; Govorushko, 2019), measures of heat effects on individuals may tell us little about effects on colonies and populations.

**FIGURE 1:**
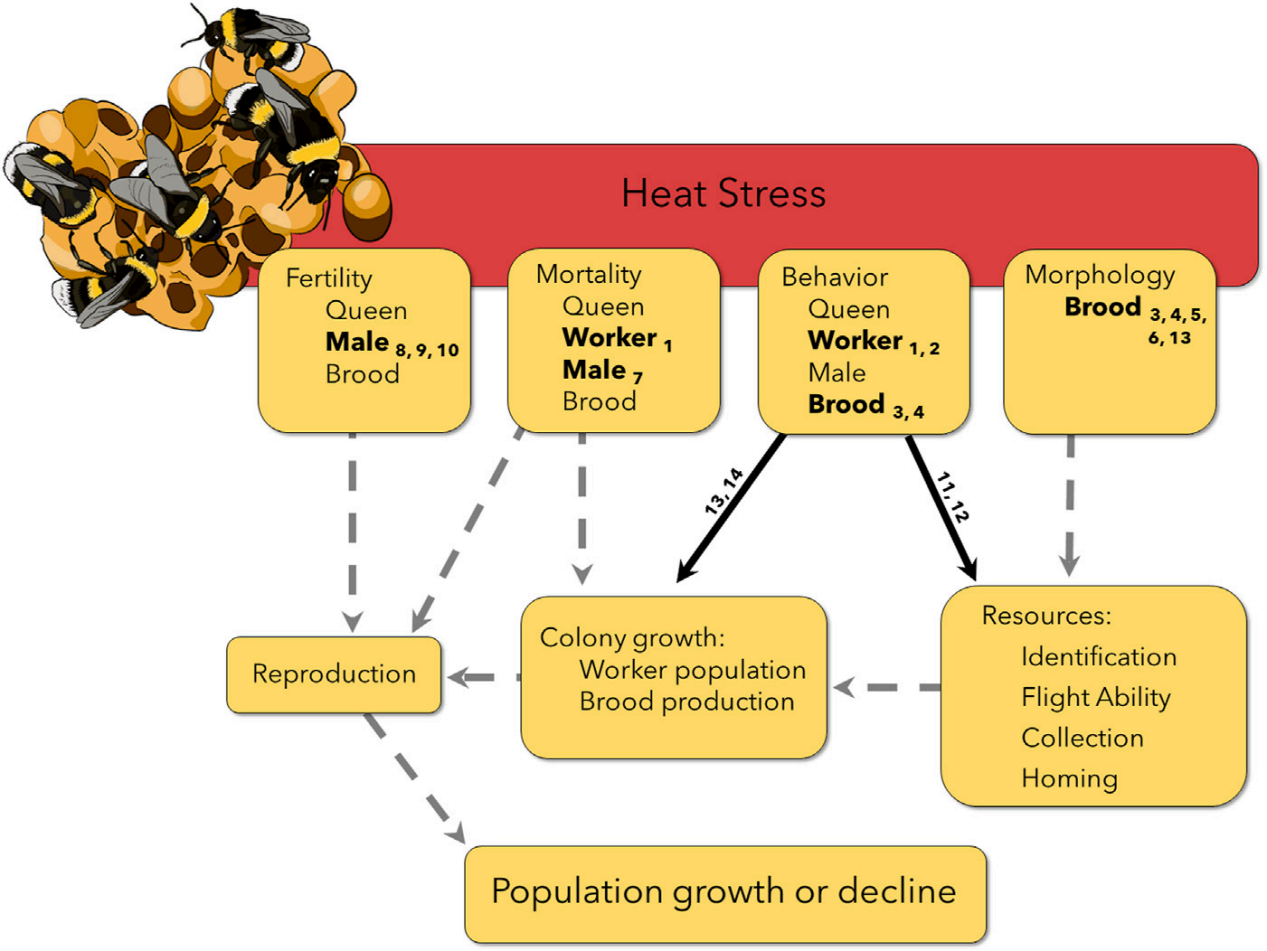
Heat stress experienced by different life stages and castes can lead to propagated effects later in the colony life cycle, potentially affecting reproduction and therefore populations persistence. Solid lines have some supporting evidence (Table 1), but still require more work on non-commercial species and in field conditions. Number subscripts correspond to the reference in Table 1. Dashed lines indicate suspected interactions and carry-over effects that have not yet been explicitly tested, highlighting the need for additional research. For example, workers exposed to heat stress were less able to learn and remember a stimulus for a sucrose reward (Gerard et al., 2022b), indicated in the figure as an effect on behavior. In a field setting, this could potentially affect a worker’s homing ability or identification of resources, leading to mortality on a foraging flight or fewer resources returning to the colony. Both carry over effects could decrease colony growth and lead to fewer available resources to invest in reproduction. However, without additional research, we do not know if a colony is able to compensate for heat stressed individuals, for example, by recruiting workers from other tasks to ensure resource collection remains high.

Bumble bees (genus *Bombus*) have experienced well-documented declines in Europe and North America that are clearly linked to increasing temperatures (Sirois-Delisle and Kerr, 2018; Soroye et al., 2020). Recent work suggests little variation among bumble bee populations or species in CT_max_ (Oyen et al., 2016; Oyen and Dillon, 2018; Pimsler et al., 2020; Christman et al., 2022; Gonzales et al., 2022), as well as a lack of acclimation capacity in maximum thermal tolerance after a heat wave event (Oyen and Dillon, 2018; Quinlan et al., 2023; Sepúlveda and Goulson, 2023). Yet some species appear to be benefiting from warming climates whereas many others are in decline (Jackson et al., 2022). Heat exposure below CT_max_ may therefore play an important role in differential responses to climate warming (Martinet et al., 2021a). Further, effects of heat exposure are almost universally measured on female workers (but see Maebe et al., 2021; Campion et al., 2023; Martinet et al., 2021b; Bretzlaff et al., 2023), but bumble bees are eusocial insects that live in colonies, so effects of heat exposure must be considered in the context of the colony life cycle. Queen bumble bees emerge in spring and start new colonies (usually underground), with worker populations that grow throughout summer. Colonies produce new queens and males in the fall; after mating, males, workers, and the old queen perish, with newly fertilized queens overwintering underground until the cycle begins again the following spring (Goulson, 2010). Therefore, effects of heat exposure on female workers are only relevant when they alter the colony’s success at producing reproductives in the fall. Even then, new queens and males may be compromised by heat exposure, further threatening bumble bee populations. A mechanistic understanding of climate change effects on bumble bee populations therefore requires consideration of effects of heat exposure on diverse traits that together determine whether colonies produce new queens that successfully mate and produce colonies the following spring (Figure 1). Here, we focus on four core traits (mortality, behavior, morphology, and fertility), for which we explore the potential importance of sub-lethal heat, carry-over effects, and colony compensation in mediating the impacts of heat exposure. Heat exposure that does not immediately kill the animal is often considered to be sub-lethal, but delayed mortality is rarely considered. Further, heat exposure at one life stage (though not lethal) may still alter key traits of a subsequent life stage, thereby causing carry over effects later in the colony lifecycle. Finally, colonies may compensate for lethal, sub-lethal, and carry-over effects of heat exposure on individuals to preserve reproductive output, potentially mitigating these effects.

## Mortality

Bumble bees are not only incapacitated, but often die after exposure to CT_max_, which often exceeds 50°C for these animals (Oyen and Dillon, 2018). But exposure to far less extreme temperatures can also shorten bumble bee lifespan (Martinet et al., 2015; Kuo et al., 2023), likely due to accumulation of heat injury that is either irreversible or cannot be repaired quickly enough (Jørgensen et al., 2021; Ørsted et al., 2022). Unfortunately, to our knowledge, no studies have systematically investigated the durations of heat exposure at different temperatures that lead to premature mortality in bumble bees. Workers may regularly encounter stressfully hot temperatures below CT_max_ while foraging, but it is unclear if such exposure results in premature death. Loss of workers may compromise brood care and resource collection if worker numbers are limiting. When foragers are removed, colonies will sometimes compensate by recruiting additional foragers or increasing foraging activity (Biella et al., 2019; Gerard et al., 2023a). Colony success could, therefore, be unaffected by loss of workers due to heat exposure. However, if compensation is insufficient, fewer workers could lead to a reduction in the quantity and quality of resources collected, dramatically altering colony growth, reproductive output, and sex ratio (Muller and Schmid-Hempel, 1992; Pelletier and McNeil, 2003; Crone and Williams, 2016; Lanterman and Goodell, 2018; Vaudo et al., 2018). In this way, increased heat exposure of workers, though not immediately lethal, could carry-over to affect population persistence (Figure 1).

Heat exposure may also kill developing brood. Underground nests likely buffer colony brood from temperature extremes in the near term but may also serve as chronically warm traps in the long term (Woods et al., 2021) with unknown effects on brood viability. Nest thermoregulation by workers may further buffer brood (Gardner et al., 2007), but at a cost–resources allocated for worker thermoregulatory behaviors may be taken away from brood production (Baudier and O’Donnell, 2017). Bretzlaff et al. (2023) found CT_max_ values to be significantly lower in larvae, as well as a 22%–36% increase in colony energy expenditure at ambient temperatures above 30°C, further reinforcing the importance and high costs of nest thermoregulation. To our knowledge, effects of sub-lethal heat on delayed mortality of bumble bee eggs, larvae, and pupae are not known. But, given their development in a thermoregulated underground nest, these life stages may have narrow thermal tolerance breadths (as do tropical relative to temperate insects; Deutsch et al., 2008), making them more susceptible to occasional temperature spikes. As with workers, effects of heat on brood mortality could propagate to alter colony success, particularly if the affected brood are reproductives.

Beyond workers and brood, heat exposure could also profoundly affect mortality of queens outside the nest (fall, winter and spring) and of males but this has not been studied, to our knowledge.

## Behavior

Beyond mortality, heat stress can have striking effects on worker behavior, with potential carry-over effects on colony success (Figure 1; Table 1). In artificial foraging environments, with limited space and controlled flower availability, heat stress decreased the number of foraging trips, of recruited foragers, and of successful foraging attempts (Gerard et al., 2022a; Hemberger et al., 2022). However, Greenop et al. (2020) found no change in foraging behavior after the colony box was heated to 31°C, but only recorded foraging on a single plant placed in a foraging chamber for 25–30 min. Heat-exposed bees also had altered resource preferences, bringing less pollen back to the colony (Kuo et al., 2023). These changes in foraging behavior may in part reflect the effects of heat on memory. Heat-stressed worker bumble bees exposed to 32°C were significantly worse at learning to associate a colored light with a sucrose reward and were unable to remember that association after an hour compared to bees at 25°C (Gerard et al., 2022b). Others have also found that heat-stressed workers were more likely to respond abnormally to stimuli such as honey odor, UV light, and air puffs (Perl et al., 2022). This suggests that foragers experiencing heat stress could have trouble remembering quality foraging locations or even fail to navigate home. However, minor foraging errors may actually be beneficial for discovering and exploiting new food sources (Evans and Raine, 2014), and it is unclear if or when bees recover from these memory and learning deficits. Given the importance of resources, particularly pollen, for brood development and colony growth, these short-term shifts in forager behavior caused by heat exposure could affect long-term colony success.

**TABLE 1:** The effects of heat exposure on bumble bees have focused primarily on direct effects on workers, with some data available for males and for whole colony responses. Almost all studies have compared responses at two temperatures and none, to our knowledge, have directly investigated how effects of heat stress on one or more life stages or castes may have carry-over effects on colony success. The data in this table are correlated with numbers in Figure 1.

Life stage	Effect	Species	Conditions	Findings	Reference	Number in Figure 1
Workers	Mortality	*B. eximius*	Workers kept in laboratory at 26°C, 32°C, or 35°C for 5 or 7 days.	100% mortality after 7 days at 35°C.	Kuo et al., (2023)	1
	Behavior	*B. terrestris* (commercial)	Bees were exposed to either 32°C or 25°C and trained to associate a colored light with a sucrose reward.	Bees at 32°C were worse at learning to associate colored light with sucrose reward and unable to remember it after an hour.	Gerard et al., (2022b)	2
		*B. eximius*	Workers kept in laboratory at 26°C, 32°C, or 35°C for 5 or 7 days.	Heat treated bees (32°C) had decreased appetite, energy metabolism, and wingbeat frequency, and collected less pollen and flew shorter distances in a flight chamber.	Kuo et al., (2023)	1
Brood	Behavior	*B. terrestris* (commercial and wild)	After rearing in laboratory at 33°C or 27°C, colonies were kept in a temperature-controlled foraging room with real flowers.	Commercial colonies reared at higher temperatures had fewer foragers that performed fewer foraging trips. Workers from wild colonies did not leave the colony.	Gerard et al., (2022a)	3
		*B. terrestris* (commercial)	Reared in laboratory at 33°C or 26°C for the latter portion of development (11–20 days).	Bees reared at higher temperature had more “unexpected” responses to stimuli (UV light, white light, white sugar water, quinine, honey odor, brown sugar water, air puff).	Perl et al., (2022)	4
	Morphology	*B. terrestris* (commercial)	Reared in laboratory at 33°C or 25°C for entire larval development.	Higher developmental temperature led to smaller body size and shorter antennae in adult workers. Additionally, higher temperatures led to tongue length relative to body size to be closer to isometric.	Gerard et al., (2023b)	5
		*B. terrestris* (commercial and wild)	Reared in laboratory at 33°C or 25°C for entire larval development.	Workers reared at high temperatures had overall broader and longer wings, with no change in adult body size.	Gerard et al., (2022a)	3
		*B. terrestris* (commercial)	Reared in laboratory at 33°C or 25°C for entire larval development.	Decreased wing centroid size and increase in wing size asymmetry in heat stressed individuals.	Gerard et al., (2018)	6
		*B. terrestris* (commercial)	Reared in laboratory at 33°C or 26°C for the latter portion of development (11–20 days).	No effect on body or organ size.	Perl et al., (2022)	4
Males	Mortality	*B. alpinus*	Adults kept at 40°C until heat stupor.	An average of 50% mortality in all species except *B. lucorum* which had 26% survival. *B. lucorum* had higher heat resistance and took significantly longer to reach heat stupor.	Martinet et al., (2015)	7
		*B. balteatus*				
		*B. monticola scandinavicus*				
		*B. monticola rondoui*				
		*B. lucorum* (wild)				
	Fertility	*B. impatiens* (commercial)	Whole males held at 45°C until heat stupor for *in vivo*. Extracted seminal vesicles placed at 45°C or 22°C for 85 min for *in vitro*.	45°C for as little as 30 min decreased spermatozoa viability both *in vitro* and *in vivo*.	Campion et al., (2023)	8
		*B. terrestris*	Males held at 40°C until heat stupor.	Sperm viability decreased from 60% to 20% in *B. magnus* and *B. jonellus* after heat exposure. Cephalic labial gland structure and pheromone production was significantly affected. No impacts to fertility in *B. terrestris.*	Martinet et al., (2021a)	9
		*B. magnus*				
		*B. jonellus*				
		*B. terrestris* (commercial)	Males held at 40°C until heat stupor.	Virgin queens had no pheromone preference between males that reached heat stupor at 40°C vs. controls, nor did mating with treated males impact copulation behavior or brood composition.	Przybyla et al., (2021)	10
Whole Colony	Behavior	*B. impatiens* (commercial)	Plant nectar production and foraging behavior observed in enclosure at 25°C or 35°C.	Foragers in 35°C had the same number of foraging bouts as 25°C foragers, however they spent more time at the entrance attempting to forage but not leaving.	Hemberger et al., (2022)	11
		*B. terrestris* (commercial)	Colonies placed at 25°C or 31°C in enclosure with faba bean plants.	No impact to foraging behavior, but beans exposed to heat stressed bees had a lower proportional pod set.	Greenop et al., (2020)	12
	Morphology	*B. terrestris audax* (commercial)	Colony kept in laboratory incubator for 2 months at 33°C or 25°C.	Smaller worker body size when larvae developed under warm conditions.	Guiraud et al., (2021)	13
	Colony Growth	*B. terrestris audax* (commercial)	Colony kept in laboratory incubator for 2 months at 33°C or 25°C.	More queens were produced in 33°C colonies, but the rest of the colony composition was not different.	Guiraud et al., (2021)	13
		*B. terrestris* (commercial)	Long and short stress periods at 33°C, with 26°C as the control. Heat stress was also combined with nutritional stress and small or large colonies.	Nutritional and heat stress decreased colony development and lowered investment in brood production. These effects were more noticeable in small colonies but access to high quality resources lessened those effects.	Vanderplanck et al., (2019)	14

## Morphology

For insects and many other invertebrates, temperature profoundly affects development rate (Gillooly et al., 2002; Dillon and Frazier, 2013) and body size, with hotter temperatures generally leading to faster development and smaller adult body size (the temperature-size rule; Atkinson, 1994). For *Bombus terrestris*, larval development at 33°C resulted in smaller than normal adult workers (Guiraud et al., 2021; Gerard et al., 2023b). However, this is not the case for all studies and therefore degree and timing of heat stress may lead to differing responses in final adult size (Gerard et al., 2022a; Perl et al., 2022). Nevertheless, shifts in worker body size distribution due to heat exposure could have carryover effects on subsequent colony growth throughout a season. Larger workers have larger foraging ranges (Greenleaf et al., 2007; Kendall et al., 2022) and increased flight endurance (Kenna et al., 2021), both of which facilitate their ability to collect more resources (Kerr et al., 2019), thereby potentially contributing more to colony growth. Without compensation at the colony level, heat exposure could lead to smaller workers which gather fewer resources, limiting colony growth and, by extension, number of reproductives (e.g., Crone and Williams, 2016).

Higher developmental temperatures also resulted in shorter antennae, tongue lengths closer to isometry, and smaller wings (Gerard et al., 2018; Gerard et al., 2022a; Gerard et al., 2023b). Changes in antennae could affect a worker’s ability to identify quality resources (Howell and Alarcón, 2007), and tongue length has been linked to which flowers are visited (Miller-Struttmann et al., 2015), so these morphological differences due to heat exposure could affect resource collection. However, larger bees lose heat less quickly (Stevenson, 1985; Stone and Willmer, 1989; a benefit in colder environments) and carrying larger pollen loads increases thoracic temperatures (Stone and Willmer, 1989; Naumchik and Youngsteadt, 2023), therefore larger workers are conceivably more at risk of overheating while foraging.

Given demonstrated effects on worker morphology, larval heat exposure likely also alters morphology of adult males and new queens, potentially affecting their subsequent fitness. For example, males leave the colony early so must endure temperature fluctuations outside the nest; smaller males may be more vulnerable to these hot and cold extremes (Stone and Willmer, 1989). Larger queens are often more likely to survive winter likely in part because they can accumulate larger fat reserves and burn through them more slowly (Owen, 1988; Vesterlund et al., 2014; Keaveny and Dillon, 2022). Heat exposure during development could therefore result in fewer queens surviving to the next spring.

Morphological changes resulting from heat stress could have important carry-over effects that are largely unstudied (Figure 1), with the few existing studies all for a single species (*Bombus terrestris*). Even selection of the appropriate temperatures for developmental heat stress experiments is difficult because we lack measurements of wild nest temperatures under normal and heat wave conditions (but see Gradišek et al., 2023).

## Fertility

Male bumble bees leave the colony soon after emerging as adults and never return to the protection of the nest. It is therefore highly likely that they will be exposed to temperature extremes while searching for a mate. Laboratory experiments have found that exposing adult males to heat stress significantly reduces sperm viability in several bumble bee species (Martinet et al., 2021a; Campion et al., 2023). Considering the importance of male bumble bees for reproduction and that many bumble bee species are monandrous, a decrease in male fertility after heat stress could have profound implications for population growth and colony success (Figure 1). If a monandrous queen mates with a sterile male, she would be unable to produce female workers the following season. Male bumble bee sperm has been shown to be affected by heat stress both *in vitro* and *in vivo* (Campion et al., 2023), however it is still currently unknown if heat stress could also affect sperm that is stored in queen spermatheca or if heat stress affects queen ovary development. Given clear effects on worker behavior (see above), heat-stressed males may be less competitive for mates due to effects on memory and flight ability. Heat stress also alters male cephalic labial gland structure and pheromone production (Martinet et al., 2021b), with possible effects on mate recognition (Valterová et al., 2019). Future studies should consider potential effects of heat exposure on male and queen mate recognition, mating success, and subsequent brood production.

## Future avenues

Bumble bees are critically important pollinators both in natural landscapes and commercial agriculture (Ollerton, 2017; Cameron and Sadd, 2020), and population declines due to climate change are both striking and species specific (Jackson et al., 2022). As we’ve highlighted above, a better understanding of the effects of sub-lethal heat exposure on different life stages (egg, larvae, pupae, adult), of all castes (worker, male, queen), and of multiple species is a critical first step to drawing mechanistic connections between warming climates and population declines (Figure 1). This more holistic view will be similarly critical to explain and mitigate effects of climate change on other pollinators (Johnson et al., 2023) and insects more broadly (Ma et al., 2021; Harvey et al., 2023). Additionally, for organisms that live in colonies, experiments must move beyond measurements only of individual animals. Colonies may be able to compensate for lost or compromised colony members, thereby mitigating effects of heat exposure on individuals (Maebe et al., 2021). Alternatively, apparently mild effects on individuals may have unanticipated carry-over effects on colonies. Further, the vast majority of studies of heat stress effects are done in the laboratory using commercially available *B. terrestris* and *B. impatiens* (Table 1)*,* so we have little information on most wild bumble bee species (but see Gerard et al., 2022a; Martinet et al., 2015; Martinet et al., 2021b) in wild settings. This may in part reflect the difficulty in starting colonies from wild caught queens (Christman et al., 2022; Rowe et al., 2023), a challenge we’ll need to overcome if we are to better understand species-specific responses to ongoing climate change.

While beyond the scope of this review, interacting effects are also an important consideration for heat stress (see Johnson et al., 2023). Exposure to multiple forms of stress such as pesticide exposure, nutritional stress, and increased parasite load can have unexpected adverse effects (Goulson et al., 2015; Botías et al., 2021; Weidenmüller et al., 2022) and in some cases, a lack of stress (e.g., access to high quality nutrition) can lead to increased tolerance (Vanderplanck et al., 2019; Quinlan et al., 2023). It is unlikely that a colony will only experience a single stressor in its lifetime and considerations of interactions between stressors is another important area of future research.
